# *In vitro* effect of chlorambucil on human glioma cell lines (SF767 and U87-MG), and human microvascular endothelial cell (HMVEC) and endothelial progenitor cells (ECFCs), in the context of plasma chlorambucil concentrations in tumor-bearing dogs

**DOI:** 10.1371/journal.pone.0203517

**Published:** 2018-09-07

**Authors:** Michael J. Reese, Deborah W. Knapp, Kimberly M. Anderson, Julie A. Mund, Jamie Case, David R. Jones, Rebecca A. Packer

**Affiliations:** 1 Department of Veterinary Clinical Sciences, College of Veterinary Medicine, Purdue University, West Lafayette, Indiana, United States of America; 2 Center for Cancer Research, Purdue University, West Lafayette, Indiana, United States of America; 3 Angio BioCore, Indiana University Simon Cancer Center, Indianapolis, Indiana, United States of America; 4 Department of Medicine, Division of Clinical Pharmacology, Indiana University School of Medicine, Indianapolis, Indiana, United States of America; University of Texas M. D. Anderson Cancer Center, UNITED STATES

## Abstract

The objective of this study was to investigate a possible mechanism of action of metronomic chlorambucil on glioma by studying the *in vitro* cytotoxicity and anti-angiogenic effects on glioma and endothelial cells, respectively. The *in vitro* LD_50_ and IC_50_ of chlorambucil were determined using human SF767 and U87-MG glioma cell lines, human microvascular endothelial cells (HMVECs) and human endothelial colony forming cells (ECFCs). Results were analyzed in the context of chlorambucil concentrations measured in the plasma of tumor-bearing dogs receiving 4 mg m^-2^ metronomic chlorambucil. The LD_50_ and IC_50_ of chlorambucil were 270 μM and 114 μM for SF767, and 390 μM and 96 μM for U87-MG, respectively. The IC_50_ of chlorambucil was 0.53 μM and 145 μM for the HMVECs and ECFCs, respectively. In pharmacokinetic studies, the mean plasma C_max_ of chlorambucil was 0.06 μM. Results suggest that metronomic chlorambucil in dogs does not achieve plasma concentrations high enough to cause direct cytotoxic or growth inhibitory effects on either glioma or endothelial cells.

## Introduction

Gliomas are common primary brain tumors in both human and veterinary medicine. A diagnosis of a high-grade glioma carries a poor prognosis due to frequent disease recurrence [1–2]. Treatment strategies for gliomas have evolved over the last few decades; however, for patients with high-grade gliomas, there has only been modest changes in overall survival rates, even with improvements in cytoreductive surgery, radiation therapy techniques, and the addition of newer chemotherapy agents and targeted therapies [2–4].

One of the intriguing new approaches to cancer treatment is the use of relatively low, frequent dosing of chemotherapy agents, in a “metronomic” fashion to target tumor angiogenesis [5–8]. This contrasts to conventional chemotherapeutic protocols that utilize a maximum tolerated dose of drug given with prolonged breaks in between dosing to directly have an effect on tumor cells. The mechanism by which metronomic chemotherapy affects tumor angiogenesis and potentially other processes has yet to be fully elucidated. The mechanism is likely multifactorial, including targeting tumor angiogenesis through a direct effect on activated vascular endothelial cells and circulating endothelial progenitor cells, but it may also be mediated by prostaglandins, thrombospondin 1, growth factors, as well as other potential mediators of vasculogenesis [5,7–9].

An appealing aspect of metronomic chemotherapy is a lower risk of toxicity compared to standard chemotherapy [10–12]. Additionally, drugs or protocols that target tumor angiogenesis, such as metronomic therapy, should have activity against, in theory, all tumor types. In addition, previous reports have demonstrated efficacy of metronomic chemotherapy in tumors that have become resistant to conventional chemotherapy protocols, even when using the same chemotherapy agents that had shown resistance when administered in conventional dosing protocols [10,13–14].

Metronomic chemotherapy has been shown to have potential benefits in human patients with glioma [11–12,15–16]. Patient selection likely affects these results, as this therapy is more commonly utilized in patients with high grade gliomas. In these cases, disease progression occurs after a combination of surgical resection, radiation therapy, and standard chemotherapy dosing with temozolomide, which are the common therapies utilized depending on tumor grade [1,3,17–18]. Conventional chemotherapy options for glioma are limited due to the availability of drugs that penetrate the blood brain barrier (BBB). However, since the blood vessels are a component of the blood brain barrier, targeting glioma vasculogenesis may eliminate the necessity to select for a drug that crosses the BBB. Targeting tumor vasculogenesis in glioma, especially high-grade glioma, is a reasonable objective as these are commonly vascular tumors [19–20].

Chlorambucil is an alkylating agent that has been reported to inhibit neovascularization via its effect on circulating endothelial progenitor cells, induction of endothelial cell apoptosis, and inhibition of endothelial cell migration [21]. Chlorambucil has been used in the treatment of several tumor types in canine patients, with reported activity against chronic lymphocytic leukemia, lymphoma, and myeloma [22–24]. Metronomic chlorambucil has been reported to be effective with minimal toxicity in dogs with multiple types of naturally occurring cancers, at a dose of 4 mg m^-2^ per day [10], although prolonged use, especially beyond a year, or higher dosing at 8 mg m^-2^, has been associated with chronic myelosuppression in some dogs [25].

The purpose of this study was to investigate putative mechanisms of action of metronomic chemotherapy with chlorambucil against canine glioma cell lines, for use in advancing our understanding of the potential use of metronomic chlorambucil in veterinary neurooncology, and potential application to human neurooncology. There were three specific objectives. The first objective was to determine the *in vitro* median lethal dose (LD_50_) and the concentration that inhibited proliferation by 50% (IC_50_) of chlorambucil using two human glioma cell lines, SF767 and U87-MG. This establishes the concentrations of chlorambucil that would be necessary to directly inhibit proliferation of these cells. The second objective was to determine the effects of chlorambucil in inhibiting proliferation and tube formation in two human endothelial cell lines, human microvascular endothelial cells (HMVECs) and human progenitor derived endothelial colony forming cells (ECFCs). The final objective was to perform *in vivo* pharmacokinetic analyses of single-dose chlorambucil in dogs, to determine if the peak chlorambucil concentrations achieved would approximate the anti-proliferative and anti-angiogenic concentrations found *in vitro*. The hypothesis, based on previously reported clinical response, was that the IC_50_ and LD_50_ of endothelial cells would be lower than that for the glioma cells, and would approximate the peak concentrations achieved *in vivo*.

## Materials and methods

All animal work was approved by the Purdue University Institutional Animal Care and Use Committee (Protocol 11–029, approved June 2, 2011).

### Cell lines and culture conditions

SF767 (temozolomide resistant) and U87-MG (temozolomide sensitive) human glioma cell lines (*In Vivo* Therapeutics Core of the Indiana University Melvin and Bren Simon Cancer Center, Indianapolis, IN, USA) were grown as monolayers in 75 cm^2^ tissue culture flasks in Iscove’s Modified Dulbecco’s Medium (IMDM), supplemented with 10% fetal bovine serum (FBS), 1% glutamine, 100 IU/mL penicillin and 100 μg/mL streptomycin mixture. Cell cultures were maintained at 37^0^ C in a humidified 5% CO_2_ atmosphere, and sub-cultured three times each week. All assays using glioma cell lines were done independently in triplicate, using cell cultures at low passages. HMVECs and umbilical cord blood derived ECFCs (Angio BioCore [ABC]), Indiana University Melvin and Bren Simon Cancer Center, Indianapolis, IN, USA) were seeded on 75 cm^2^ tissue culture flasks pre-coated with type 1 rat tail collagen in complete Endothelial Cell Growth Medium (EGM-2) for passage.

### SF767 and U87-MG cell proliferation

Sulforhodamine B (SRB) assay (Sigma, St Louis, MO, USA) was performed as previously described [26], to determine the concentration of chlorambucil (Leukeran®, GlaxoSmithKline, Research Triangle Park, NC, USA) resulting in 50% growth inhibition (IC_50_). Briefly, the cells were detached using a trypsin-EDTA mixture and were plated using 5x10^4^ cells per well in a flat-bottom 96 well plate. The cells were incubated for 24 hours to allow for adherence before treatment. Chlorambucil in 1% DMSO was applied in the following concentrations: 1000, 500, 100, 10, 1, 0.1 and 0.01 μM (8 wells per condition). The cells were incubated following treatment for 48 hours, at which time the cells were fixed. Cold trichloroacetic acid (10% final concentration) was gently added to each well, incubated at 4^0^ C for 1 hour, washed 5 times with distilled water, and air-dried. The fixed cellular proteins were stained using 50 μL 0.4% SRB in 1% acetic acid, and incubated at room temperature for 30 minutes. The wells were washed five times using a 1% acetic acid solution and then air-dried. Bound SRB was then solubilized using 200 μL of 10 mM Tris-base solution, and the absorbance was evaluated in a 96 well EMax Endpoint ELISA Microplate Reader (Molecular Devices LLC, Sunnyvale, CA, USA) at 490 nm. Using three independent replications, the optical densities of wells for each condition (different drug concentrations or vehicle control) were averaged, and data expressed as percent growth of control. The mean and standard deviation of the replicate analyses were graphed using Excel® (Microsoft, Corp, Redmond, WA, USA), which was then used to determine the IC_50_.

### SF767 and U87-MG cell viability

**Fig 1:**
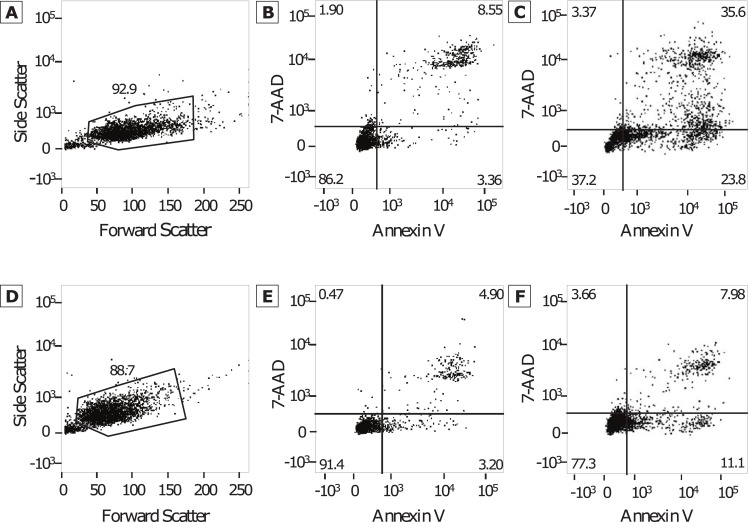
FACS analysis plots. Representative examples of plots obtained using Annexin V and 7-AAD evaluated with FACS analysis, used to determine gaiting (A and C) and evaluation of the control samples (B and D) for SF767 (A and B) and U87-MG (C and D) glioma cell lines. The percentage of early apoptotic cells (lower right quadrant) and late apoptotic cells (upper right) were then used in assessment of cytotoxicity.

Cellular apoptosis and necrosis were evaluated with the Annexin V-FITC/7-AAD kit staining protocol (BD Biosciences, Sparks, MD, USA), followed by fluorescence-activated cell sorting (FACS) analysis. The cells were detached using a trypsin-EDTA mixture and plated using 1x10^5^ cells per well in a 12 well plate. The cells were allowed 24 hours for adherence before treatment. Chlorambucil in 1% DMSO was added at each of the following concentrations: 500, 100, 10, 1, 0.1, and 0.01 μM. Cellular viability was evaluated at 4, 24, and 48 hours after exposure to chlorambucil. The supernatant, along with the cells after the addition of trypsin-EDTA mixture were collected from each well and centrifuged at 300 rpm for 5 minutes at 4^0^ C. The pellet was washed twice with 1 mL Dulbecco’s Phosphate Buffered Saline (DPBS) each time. The pellet was then re-suspended in 100 μL 1X binding buffer, and then 5 μL of Annexin V-FITC and 5 μL of 7-AAD solutions were added. The suspensions were incubated for 15 minutes at room temperature in the dark, after which 400 μL of 1X binding buffer was added. A total of 5000 events from each suspension were collected using FACS analysis with a BD FACS Canto II (Becton Dickinson and Company, Franklin Lakes, NJ, USA). The output was then analyzed using FlowJo (Version X.0.6, Tree Star, Inc, Ashland, OR, USA) to determine the percentage of healthy cells (unstained), early apoptotic cells (Annexin V-FITC positive), and late apoptotic cells (Annexin V-FITC and 7-AAD positive). Initial gating was based on Forward Scatter and Side Scatter characteristics to eliminate debris. Samples of the gating procedures for SF767 and U87-MG after 48 hours of drug exposure are provided (Fig 1). The percentages of cells in early and late apoptosis were added to determine the total percentage undergoing cell death. The mean and standard deviation of the percent cytotoxicity of the replicate analyses were graphed on Excel® using a logarithmic scale at 4, 24, and 48 hours. Using these plots, the drug concentration resulting in death in 50% of the cells (LD_50_) after 48 hours after drug exposure was determined.

### HMVEC and ECFC thymidine incorporation assay

Thymidine incorporation proliferation assays were conducted as previously described with slight modifications [27]. Cultured HMVECs and ECFCs were seeded onto a 96 well plate in EGM-2 at a density of 3000 cells per well. The cells were starved for 24 hours in Endothelial Basal Medium-2 (EBM-2) containing 2% FBS and then stimulated using Chlorambucil in 1% DMSO at the following concentrations: 1000, 500, 100, 10, 1, 0.1 and 0.01 μM for 16 hours. 1μL of tritiated thymidine was then added per well and incubated at 37^0^ C for 7 hours. The cells were then harvested and placed onto a Unifilter 96-well plate and read on a plate reader for β emission (Perkin Elmer, PerkinElmer, Inc, Waltham, MA, USA). Assays were performed in quadruplicate.

### HMVEC and ECFC matrigel tube formation assay

Matrigel assays to assess tube formation were conducted as previously described with slight modifications [28]. Briefly, early passage (i.e. 2–3) HMVECs and ECFCs were starved overnight and then seeded onto a 96-well tissue culture plate coated with 45 μL of Matrigel (BD Biosciences, Sparks, MD, USA) at a cell density of 7500 cells per well. Chlorambucil was tested in 1% DMSO at the following concentrations: 1000, 500, 100, 10, 1, 0.1 and 0.01 μM. Cells were observed every 2 hours by visual microscopy with an inverted microscope at 40X magnifications for tube formation. Wells were quantified at 8 hours post plating. Assays were performed in triplicate.

### Pharmacokinetic sampling

The dog studies were conducted following the guidelines and approval of the Purdue Animal Care and Use Committee (Protocol 11–029, approved June 2, 2011), and informed consent was obtained from all owners prior to participation. The participating dogs were client-owned pet dogs who presented to the Purdue University Veterinary Teaching Hospital for treatment of their naturally-occurring cancer. As this study aim was exclusively to acquire PK data, tumor type was irrelevant, and dogs were eligible for enrollment for any tumor type (not specifically brain tumor), provided they were treatment naïve for chlorambucil and previously scheduled to begin a metronomic chlorambucil treatment protocol. The 5 participating dogs received oral chlorambucil at 4 mg m^-2^ per day as part of their cancer therapy. Plasma samples were obtained at 0 (prior to administration of the first dose of chlorambucil), 5, 10, and 30 minutes, and then 1, 2, 4, and 8 hours after the first dose. The mean and range of the half-life (t_1/2_), time to peak serum concentration (t_max_), peak serum concentration (C_max_), area under the curve (AUC), and clearance were determined.

### Quantification of plasma chlorambucil

Chlorambucil was quantified in dog plasma and urine by internal standardization, protein precipitation with acetone, and HPLC-MS/MS (Thermo TSQ Quantum Ultra, Thermo Scientific, Waltham, MA, USA). Chlorambucil and melphalan, the internal standard, were separated on a C18 4.6 X 100 mm column (Phenomenex Onyx Monolithic, Torrance, CA, USA) using a linear gradient mixing the mobile phases. Mobile phase A was acetonitrile: 0.1% formic acid (20:80, v/v) and mobile phase B was acetonitrile: 0.1% formic acid (80:20, v/v). The linear gradient started with 100% mobile phase A at the time of injection and was 100% mobile phase B at 6 minutes. The mass spectrometer utilized an electrospray ionization probe and was run in positive mode. The selected reaction monitoring (SRM) Q1/Q3 (m/z) transition for chlorambucil and melphalan was 305/169 and 306/247, respectively. The lower limit of quantification was 1 ng/mL using 100 μL plasma.

### Pharmacokinetic analysis

The maximum plasma concentration (C_max_)_,_ was obtained from the data. Pharmacokinetic parameters for chlorambucil were estimated using non-compartmental methods with add-ins on Excel®. The half-life (t_1/2_), was estimated by 0.693/k_el_, where k_el_ is the elimination rate constant. The average chlorambucil concentrations (n = 5 dogs) were used to model the pharmacokinetics using PK Solver in Excel®. The data best fit a one compartmental model after an extravascular dosage (4 mg m^-2^). Estimates, including the absorption rate constant, k_a_, t_½_, the time of maximum concentration, t_max_, and k_el_, from this model were used to forecast C_max_, assuming linear conditions after single dosage administration.

## Results

### SF767 and U87-MG cell proliferation assay

**Fig 2:**
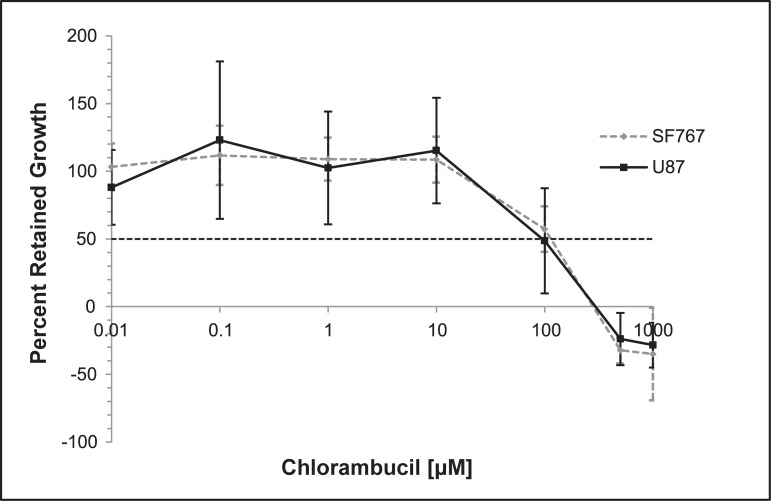
SF767 and U87-MG growth inhibition. Calculated mean percent growth inhibition of increasing concentrations of chlorambucil (0.01 to 1000 μM) against *in vitro* SF767 (gray dotted line) and U87-MG (black solid line) glioma cell lines as determined using the Sulforhodamine B assay. Data are expressed in the figure by the % of growth retained under each condition, compared to the control. The IC_50_ was then determined to be 114 μM and 96 μM for the SF767 and U87-MG glioma cell lines, respectively.

The determined IC_50_ of chlorambucil was 114 μM for SF767 cells and 96 μM for U87-MG cells (Fig 2).

### SF767 and U8-MG7 cell viability assay

**Fig 3:**
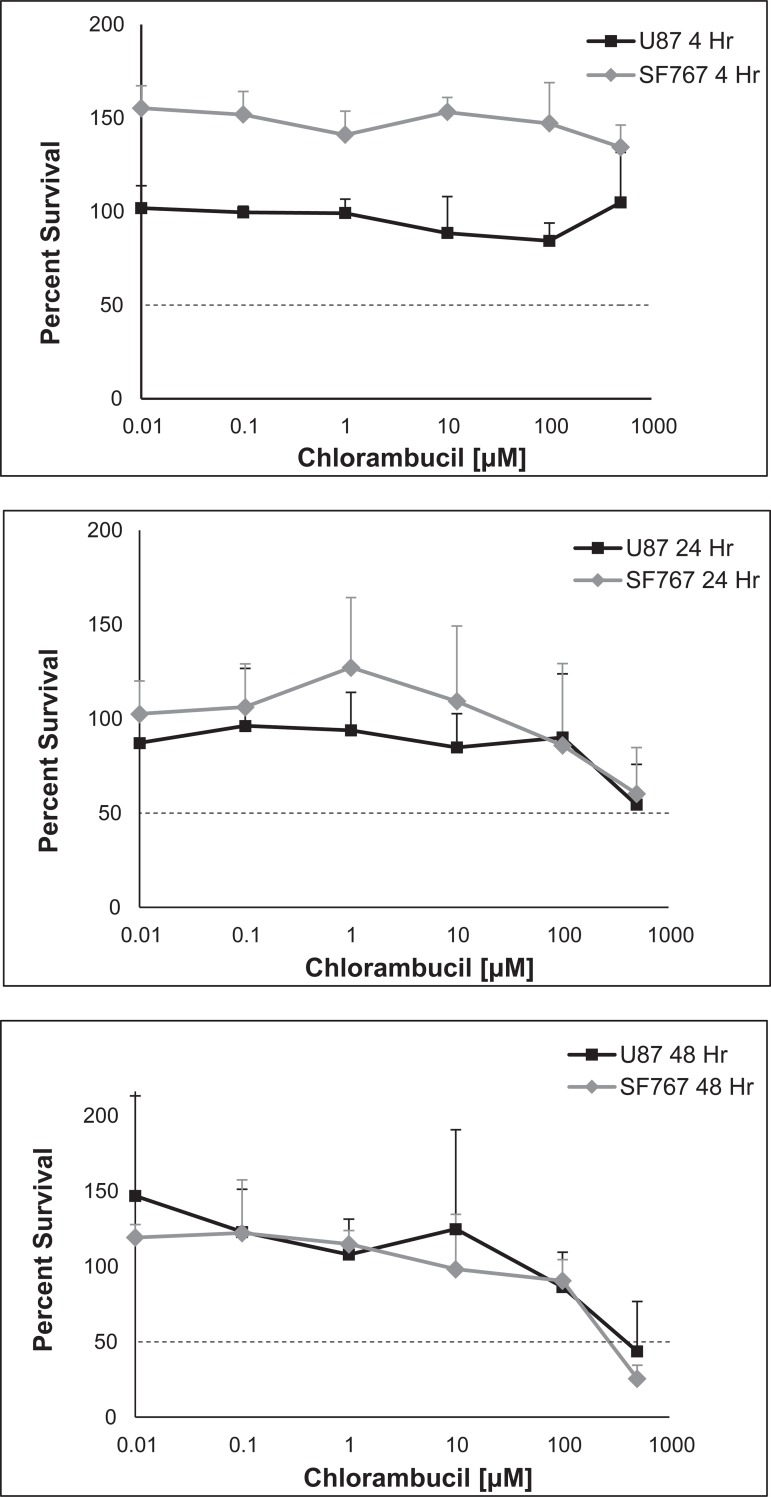
SF767 and U87-MG cytotoxicity. Average percentage survival of cells with increasing concentrations of chlorambucil (0.01 to 500 μM) against *in vitro* SF767 (gray dotted lines) and U87-MG (black solid lines) after 4 hours (A), 24 hours (B), and 48 hours (C) of exposure using Annexin V and 7-AAD and FACS analysis. Using the sum of the percentage of early apoptotic cells and late apoptotic cells as determined using FACS analysis, the LD_50_ after 48 hours was 270 μM and 390 μM for SF767 and U87-MG glioma cell lines, respectively. Note that where the % survival relative to control is greater than 100%, this is explained by insignificant cytotoxicity and natural variation of non-drug dependent cell death at early time points and low drug dilutions, in which the fraction of surviving cells in the experimental wells at lower concentrations and earlier time points was greater than that in the control wells, resulting in an initial survival greater than 100%. As incubation time and drug concentration increase, cell death becomes greater than the control and the data are more informative.

There was minimal evidence of cytotoxicity at 4 hours, which subsequently increased over 24 and 48 hours (Fig 3). At 48 hours, the LD_50_ was 270 μM for the SF767 cells and 390 μM for the U87-MG cells.

### HMVEC and ECFC proliferation and tube formation assays

**Fig 4:**
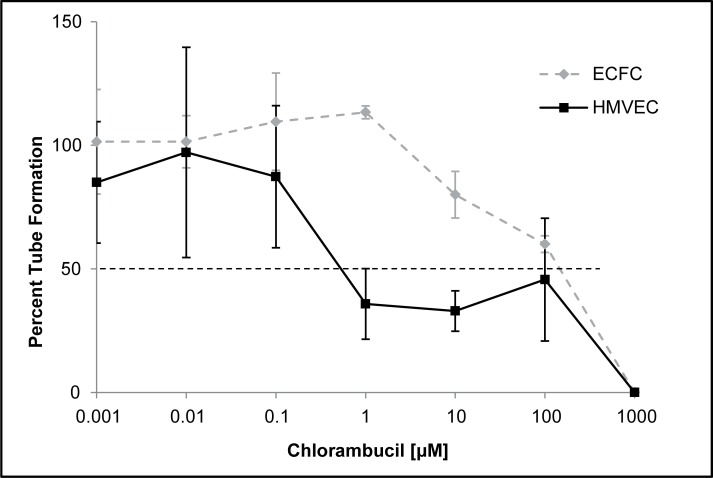
HMVEC and ECFC proliferation. *In vitro* determination of the mean percent tube formation of HMVECs (black solid line) and ECFCs (gray dotted line) against increasing concentrations of chlorambucil (0.01 to 1000 μM) using the Matrigel tube formation assay. Values are compared to control wells to provide a %. The concentration leading to a mean percent growth inhibition was 0.53 μM and 145 μM for the HMVECs and ECFCs, respectively.

The IC_50_ for inhibition of tube formation using the Matrigel assay was 0.53 μM and 145 μM for the HMVECs and ECFCs, respectively (Fig 4). When comparing the tube formation as a percentage of control, tube formation was 56% and 84% in the 1% DMSO well compared to the cells only control. Tube formation in the remaining concentrations reached 50% suppression at 1 μM and 1000 μM for the HMVECs and ECFCs, respectively. For the thymidine incorporation proliferation assays, the LD_50_ of both the HMVECs and ECFCs was 1000 μM.

### Chlorambucil pharmacokinetic profile in tumor-bearing dogs

**Fig 5:**
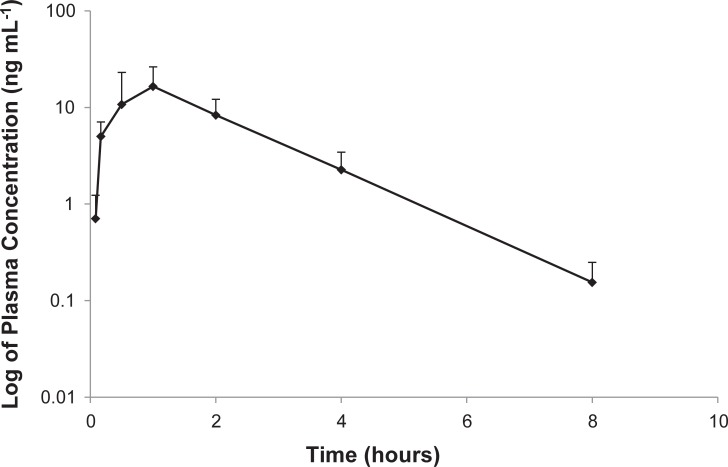
Plasma concentration of chlorambucil. Log transformation of pharmacokinetic data for 5 dogs receiving 4 mg m^-2^ per day.

A single chlorambucil dose of approximately 4 mg m^-2^ per day (actual dose range 3.80–4.76 mg m^-2^) in dogs yielded a mean t_1/2_ of 1.7 hours (range 0.9–3.6 hours) with a mean C_max_ of 20.6 ng/mL (range 4.5–31.3 ng/mL) (Fig 5). The mean C_max_ of 20.6 ng/mL is equivalent to a concentration of 0.06 μM (range of all dogs 0.01–0.09 μM). The results of the pharmacokinetic analyses are depicted in Table 1. Modeling was performed to calculate the oral metronomic dose required to reach a C_max_ that matched the results of the *in vitro* studies. The current 4 mg m^-2^ dose administered resulted in average plasma C_max_ of 0.06 μM. In order to reach a C_max_ of 0.1, 20, and 100 μM, an oral dose of 86, 1730 and 8500 mg m^-2^, respectively, would be required based on the modeling estimates.

**Table 1:** Pharmacokinetic data for 5 dogs receiving chlorambucil at 4 mg m^-2^ per day.

	Dose (mg m^-2^)	C_max_ (ng/mL)	T_max_ (hours)	t_1/2_ (hours)
**Dog 1**	4.76	31.32	0.50	1.57
**Dog 2**	3.80	4.50	0.17	3.63
**Dog 3**	4.17	17.00	1.00	1.46
**Dog 4**	4.39	28.20	1.00	0.86
**Dog 5**	4.25	21.50	1.00	0.96
**Mean (SD)**	4.27 (0.35)	20.50 (10.55)	0.73 (0.38)	1.70 (1.12)

## Discussion

As expected, the concentration of chlorambucil necessary to inhibit human endothelial cell proliferation was generally lower than the concentration necessary to inhibit human glioma cell proliferation and cause direct cytotoxicity. The HMVECs and ECFCs differed in their response to chlorambucil, which may be due to differences in maturity of endothelial cell types. HMVECs are mature endothelial cells collected from the microvasculature of the lung whereas the cord blood derived ECFCs contain high numbers of immature progenitor cells that contribute to de novo vessel formation [27]. Furthermore, it has been previously reported that ECFCs have a higher proliferative capacity compared to endothelial cells of other, more mature, origins [29]. Surprisingly, the concentrations of chlorambucil required to inhibit the proliferation and tube forming activity of either type of endothelial cells *in vitro* was much higher than chlorambucil concentrations achieved *in vivo* in tumor-bearing dogs. The previously reported clinical effect using metronomic chlorambucil in dogs, in which the reported dose resulted in a remission rate of 4 of 36 (11%) dogs, and stabilized the disease in 17 of 36 (47%) dogs, does not correlate with the disparity between plasma concentration and the concentrations necessary to inhibit human endothelial cell proliferation [10]. Based on pharmacokinetic modeling, the oral chlorambucil dosages necessary to attain a C_max_ appropriate for directly affecting endothelial cells in angiogenesis would be > 8500 mg m^-2^, which is not biologically feasible in the dog. This would suggest that chlorambucil acts through mechanisms other than those tested *in vitro*. These could include cell-cell interactions that enhance either anti-angiogenic or cytotoxic effects that were not elucidated in pure cell line cultures, such as paracrine effects, or co-factors from circulating tumor cells. There has also been consideration recently that metronomic chemotherapy may elicit a pro-immunogenic effect through proliferation of specific T-cell responses and NK cells, which is specific to the low drug dosages used in metronomic chemotherapy, and the opposite effect of the leukocyte suppression elicited by MTD regimes [30].

It is acknowledged that the *in vitro* assays in this study only assesses direct effects on endothelial cell proliferation and tube formation, and that other proposed anti-angiogenic mechanisms of metronomic chlorambucil would not be detected. This provides support for other proposed mechanisms for metronomic chemotherapy on angiogenesis, and other processes playing a larger role in the beneficial effects seen clinically with metronomic chlorambucil [10,14], though a clear mechanism of action of metronomic chlorambucil has not been established with the results of the current study. Although gaps in the understanding of metronomic chlorambucil for CNS tumors exist, a pilot study may further elucidate the mechanism of action and determine preliminary efficacy. Tumor biopsies or surgical resection could be obtained at a specific time point after administration of chlorambucil, in order to determine tissue drug concentration. This would help elucidate whether tissue concentrations were supportive of direct cytotoxic effect, or not, and could be matched with the *in vitro* data presented here, along with clinical outcome data. This information also may inform whether additional *in vitro* co-culture studies may further elucidate the mechanism of action. Further studies could also include effects on other mediators of vasculogenesis, endothelial progenitor cells, thrombospondin-1, as well as effects on regulatory T cells [8–9,31].

Another point that must be considered is whether canine and human endothelial cells could respond differently to chlorambucil. There could be species differences regarding the sensitivity of endothelial cells against chlorambucil, as well as differences in sensitivity of endothelial cells within the tumors compared to that of healthy tissues. Dog endothelial cell cultures were not available at the time of our experiments, thus human endothelial cell cultures were used in this study. However, in a mouse study, anti-angiogenic effects were only observed with 400 μM chlorambucil, while plasma concentrations ranged from 0.01 to 0.09 μM [21]. The concentration of chlorambucil causing inhibition of vasculogenesis in the mouse embryonic experiment was similar to the concentrations that inhibited angiogenesis using human endothelial cells in our current study.

When humans have received doses of chlorambucil at or approaching the maximum tolerated dose (MTD), the peak plasma concentrations (3.6 μM) approach a concentration that would inhibit proliferation in HMVECs [32]. It is interesting that due to relatively high bioavailability of chlorambucil in humans (70%) that the MTD in humans (6 mg m^-2^) is only slightly higher than the metronomic chemotherapy dosing in dogs, but achieves more than a tenfold higher peak plasma concentration [33,34]. Although it is intriguing to consider that MTD chlorambucil may have anti-angiogenic effects, the goal is to determine effective metronomic dosing that will have less risk of toxicity than MTD dosing.

Metronomic chemotherapy and other targeted therapies for tumor vasculogenesis are currently being explored for human patients with high-grade glioma, especially upon recurrence of disease. There are a limited number of reports evaluating the effects of metronomic chemotherapy, most commonly temozolamide, on patients with glioma. These studies utilized various metronomic chemotherapy drug protocols in patients with recurrent malignant gliomas, with some demonstrating improvement in progression free survival and some showing no benefit [11–12,15–16,35]. Patient selection likely contributes to the variability in these results. Most of these reports include patients that have disease recurrence following surgical resection, radiation therapy, and chemotherapy prior to the use of metronomic therapy, thus suggesting a more aggressive disease. The differences in response to treatment could also be attributed to the metronomic chemotherapy regimen that is utilized.

More research is needed in order to elucidate the actual benefit of metronomic chemotherapy for brain tumors, and which drug, or drug combination, is most beneficial. Work in dogs could prove extremely valuable in this research. Canine glioma is an accepted model for human glioma, and more closely mimics the human condition than xenograft models [36–38]. High grade gliomas in both humans and dogs are spontaneous tumors, heterogenous, infiltrative, and have many similar histopathologic findings and genetic alterations [36–38]. Although comprehensive comparisons of genetic and immunological characteristics are not yet available, similarities of significance that have been identified include chromosome copy number aberrations (gains or losses among several chromosomes) [38, 39], neovascularization [40], and endothelial/microvascular proliferation [40], while key differences include mutagenesis of IDH1 and IDH2 [41]. Of importance to further study in this model is the tumor tissue penetration of chlorambucil. It is known that in normal rat brain the brain tissue:plasma ratio of chlorambucil is 0.021, indicating poor penetration of the BBB [42]; however, this may not represent the concentration in tumor tissue.

Metronomic chemotherapy with chlorambucil has been demonstrated to be efficacious and well tolerated when used to treat multiple types of cancer in dogs [10,14,25]. In these studies, the majority of side effects were classified as grade 1–2 gastrointestinal toxicity, with some grade 1–2 thrombocytopenia and lethargy, according to the Veterinary Co-operative Oncology Group [43,25]. More severe adverse events, such as persistent thrombocytopenia or severe lethargy, were rarely reported [25].
